# Electrophysiological modulation of pain‑related sodium channels by cannabinoids: a systematic review

**DOI:** 10.1186/s42238-026-00436-6

**Published:** 2026-04-15

**Authors:** Samuel Gonçalves Castro, Sarah Eduarda Silva, Julio Cesar Moreira Brito, Alessandra Matavel

**Affiliations:** Research and Development Division, Ezequiel Dias Foundation (FUNED), 80 Conde Pereira Carneiro St, Belo Horizonte, MG 30510-010 Brazil

**Keywords:** Sodium channel, Na_v_, Nociception, Cannabidiol, Phytocannabinoids

## Abstract

**Background:**

Voltage-gated sodium channels regulate dorsal root ganglion excitability and are critical for nociceptive transmission. Cannabidiol (CBD) and related phytocannabinoids have been proposed to modulate these channels, offering potential non-opioid analgesic strategies. This systematic review evaluated their in vitro effects on pain-related sodium channels, focusing on electrophysiological and biophysical mechanisms.

**Methods:**

Following PRISMA guidelines, Embase^®^, LILACS^®^, PubMed^®^/MEDLINE^®^, and Scopus^®^ were searched using the SPIDER strategy. Eligible studies evaluated CBD or its derivatives on voltage-gated sodium channels Na_v_1.3, Na_v_1.6, Na_v_1.7, Na_v_1.8, and Na_v_1.9 through electrophysiological assays. Data extraction and analysis were conducted independently by two reviewers, with inter-rater agreement assessed by Cohen’s kappa. Seven studies met the inclusion criteria.

**Results:**

CBD consistently inhibited sodium currents with IC₅₀ values in the low micromolar range (2–3.3 µM), reduced action potential firing, induced hyperpolarizing shifts in steady-state inactivation, and delayed recovery from inactivation. Other phytocannabinoids, such as cannabigerol, cannabinol, cannabigerolic acid, and cannabidivarinic acid, also inhibited sodium channels, though with variable potency and distinct effects on channel gating.

**Conclusion:**

Cannabinoids, particularly CBD, act as non-selective inhibitors of voltage-gated sodium channels implicated in pain signaling. Their ability to stabilize inactivated channel states and reduce neuronal excitability supports their therapeutic potential in neuropathic pain. These findings highlight the relevance of phytocannabinoids as promising candidates for the development of non-opioid analgesics.

## Background

Nociception, the neural process of encoding and transmitting noxious stimuli, is primarily mediated by sensory neurons located in the dorsal root ganglia (DRG). These pseudounipolar neurons relay peripheral pain signals to the spinal cord and central nervous system through specialized ion channels and receptors that detect thermal, mechanical, and chemical insults. Chronic pain, typically defined as pain persisting beyond three months, involves persistent sensitization and long‑lasting neuroplastic changes that amplify nociceptive signaling and reduce pain thresholds. These alterations highlight its distinction from physiological nociception and underscore the relevance of ion‑channel modulation in chronic pain mechanisms (Wadhwa et al. 2025). Thus, DRG neurons play a pivotal role in initiating and modulating pain pathways, making them a central focus in studies of nociception and pain-related disorders (Basbaum et al. 2009; Gold and Gebhart 2010).

DRG neurons express a distinct repertoire of ion channels, including voltage-gated sodium channels (Na_v_1.7, Na_v_1.8, Na_v_1.9), transient receptor potential (TRP) channels, acid-sensing ion channels (ASICs), and T-type calcium channels (Ca_v_3.2), that determine excitability and pain sensitivity (Benarroch 2015). Among these, voltage-gated sodium channels (Na_v_) are central to membrane excitation and neurotransmission. In DRG neurons, Na_v_1.9 enhances subthreshold depolarizations and contributes to regulate the resting membrane potential; Na_v_1.7 amplifies these depolarizations, bringing the membrane potential closer to threshold and facilitating the initiation of action potentials; and Na_v_1.8 primarily mediates the action potential upstroke and enables repetitive firing through rapid recovery from inactivation (Alsaloum et al. 2020).

The *Cannabis* genus comprises over 140 phytocannabinoids capable of interacting with the brain’s endocannabinoid system. Among them, cannabidiol (CBD) and its derivatives modulates a wide range of ion channels and receptors, consistently reducing neuronal excitability (Patel et al. 2016; Zhang and Bean 2021; Ghovanloo et al. 2018, 2025). Although this effect is largely attributed to sodium channel blockade, CBD also inhibits potassium channels (Zhang and Bean 2021). Importantly, CBD shows no selectivity among Na_v_ channels, even across phylogenetically distant species such as humans, cockroaches, and bacteria (Ghovanloo et al. 2018). Both preclinical and clinical studies have demonstrated antinociceptive properties of CBD (Argueta et al., 2020; Villanueva et al. 2022), with a potency ranking based on IC₅₀ values for pain-related channels as follows: Na_v_1.8 (2 µM) > Na_v_1.7 (2.9 µM) > Na_v_1.6 (3.0 µM) > Na_v_1.3 (3.3 µM). The slope of the dose-response curves suggests that CBD interacts with sodium channels through multiple binding sites.

This manuscript reviews the biophysical mechanism of different cannabinoids in modulating pain-related sodium channels, including Na_v_1.3, Na_v_1.6, Na_v_1.7, Na_v_1.8, and Na_v_1.9.

## Methods

The in vitro effect of cannabidiol and its derivatives on pain-related sodium channels was assessed through a systematic review conducted in accordance with the principles outlined in the *Cochrane Handbook* (Higgins et al., 2019). The processes of article search, selection, data extraction, analysis, and interpretation were performed following the “*Preferred Reporting Items for Systematic Reviews and Meta-Analyses*” (PRISMA) guidelines (Liberati et al. 2009).

To identify relevant studies, the SPIDER strategy (Cooke et al. 2012) was employed for electronic searches, using the following criteria:

- Sample (S): Cannabidiol and its derivatives;
- Phenomenon of Interest (PI): Action on sodium channels involved in nociception;
- Design (D): Electrophysiological studies;
- Evaluation (E): Nociception outcomes (Dose response blockage and biophysical parameters of the sodium channels);
- Research type (R): In vitro studies.

This approach ensured a comprehensive and methodologically robust selection of articles, enhancing the reliability and relevance of the review findings.

A systematic search was initially conducted in five databases: Embase^®^, LILACS^®^, PubMed^®^/MEDLINE^®^ and Scopus^®^. The search strategy employed Medical Subject Headings (MeSH) such as “nociception” and “pain”, combined with specific terms including “Na_v_1.7”, “Na_v_1.6”, “cannabinoids”, among others. These descriptors were combined using the Boolean operator “AND”, as illustrated in the following example: “cannabinoids” AND “nociception” AND “Na_v_1.7”.

The search was performed on March 22, 2025, and was limited to studies published in English, Portuguese, and Spanish, with no restrictions on publication date.

In addition to electronic searches, studies classified as review articles, notes, correspondence, editorials, and letters were excluded. Further exclusions were applied based on the following criteria: (i) studies without a clearly identified biological activity assay; (ii) studies that did not involve cannabidiol (CBD) or its derivatives; (iii) studies that did not investigate Na_v_1.3 (SCN3A), Na_v_1.6 (SCN8A), Na_v_1.7 (SCN9A), Na_v_1.8 (SCN10A) and/or, Na_v_1.9 (SCN11A).

During the initial selection phase, two independent researchers (S.G.C. and S.E.S.) conducted database searches. Duplicate records were removed using Rayyan^®^, a web- and mobile-based application for systematic reviews (Ouzzani et al. 2016). Titles and abstracts of the retrieved studies were screened for eligibility based on SPIDER criteria. Studies selected through title, abstract, and keywords screening were further evaluated via full-text review.

Discrepancies were resolved through discussion with additional investigators. Inter-rater agreement was assessed using the kappa coefficient (95% confidence interval) (Landis and Koch 1977). Following a comprehensive analytical review, all relevant data were summarized in a table to facilitate critical analysis and interpretation.

## Results

### Review statistics

**Fig. 1:**
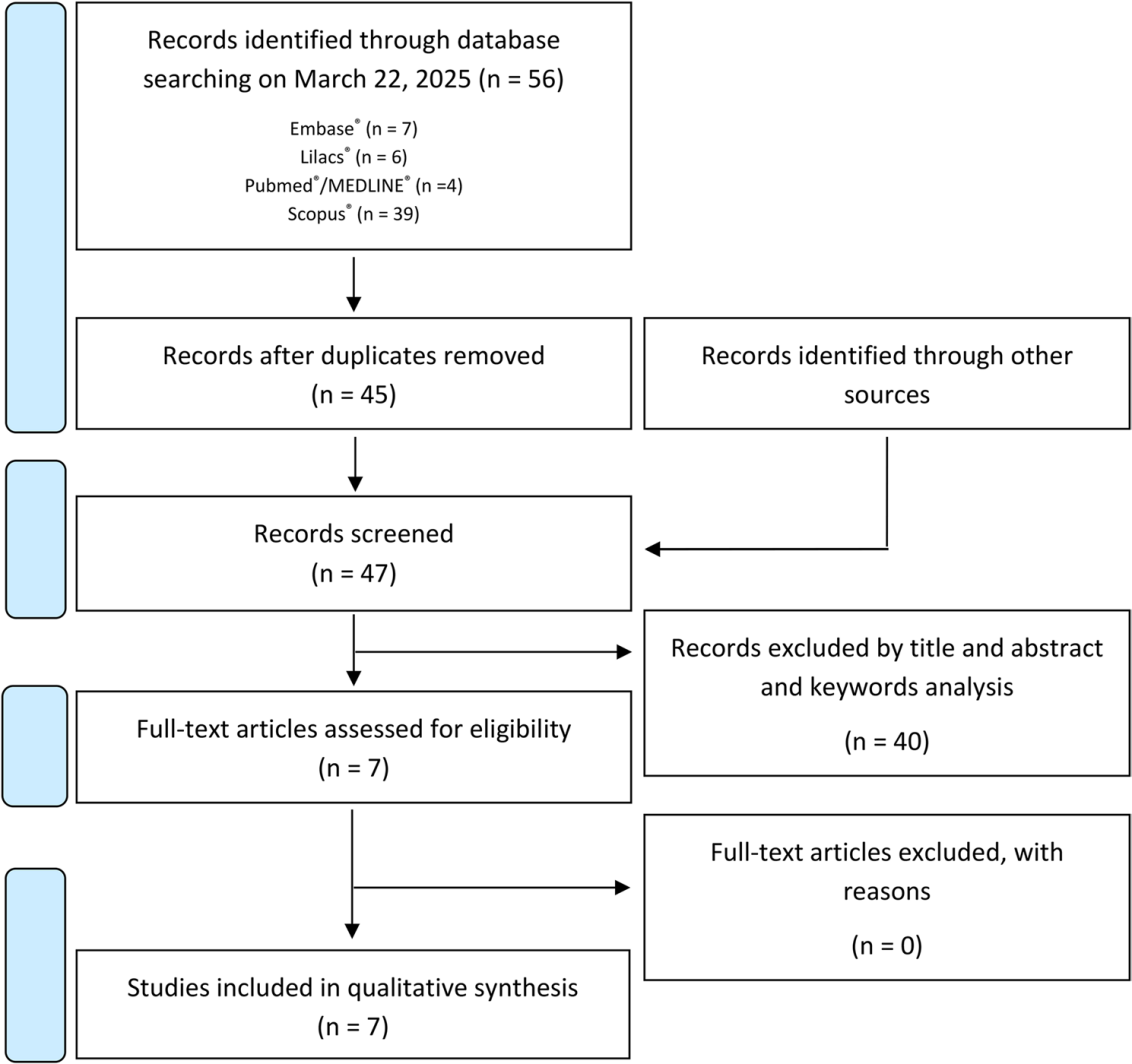
PRISMA flow diagram illustrating the study selection process. A total of 56 records were identified through database searching (Embase^®^, *n* = 7; Lilacs^®^, *n* = 6; PubMed^®^/MEDLINE^®^, *n* = 4; Scopus^®^, *n* = 39) and 2 records through other sources. After removal of duplicates, 45 records remained. Following title, abstract, and keyword screening, 7 full-text articles were assessed for eligibility. No articles were excluded at this stage, resulting in 7 studies being included in the qualitative synthesis

A bibliographic search across multiple databases yielded a total of 56 articles: 7 from Embase^®^, 6 from LILACS^®^, 4 from PubMed^®^/MEDLINE^®^ and 39 from Scopus^®^. After removing duplicates, 45 articles were screened based on titles and abstracts and with addition of 2 records identified through other sources, 7 articles met the inclusion criteria. A full-text review was subsequently performed, 7 studies were selected for qualitative analysis (Fig. 1).

Inter-rater agreement between the two researchers was classified as very strong, with a Cohen’s kappa coefficient of 0.831.

### Study characteristics

**Fig. 2:**
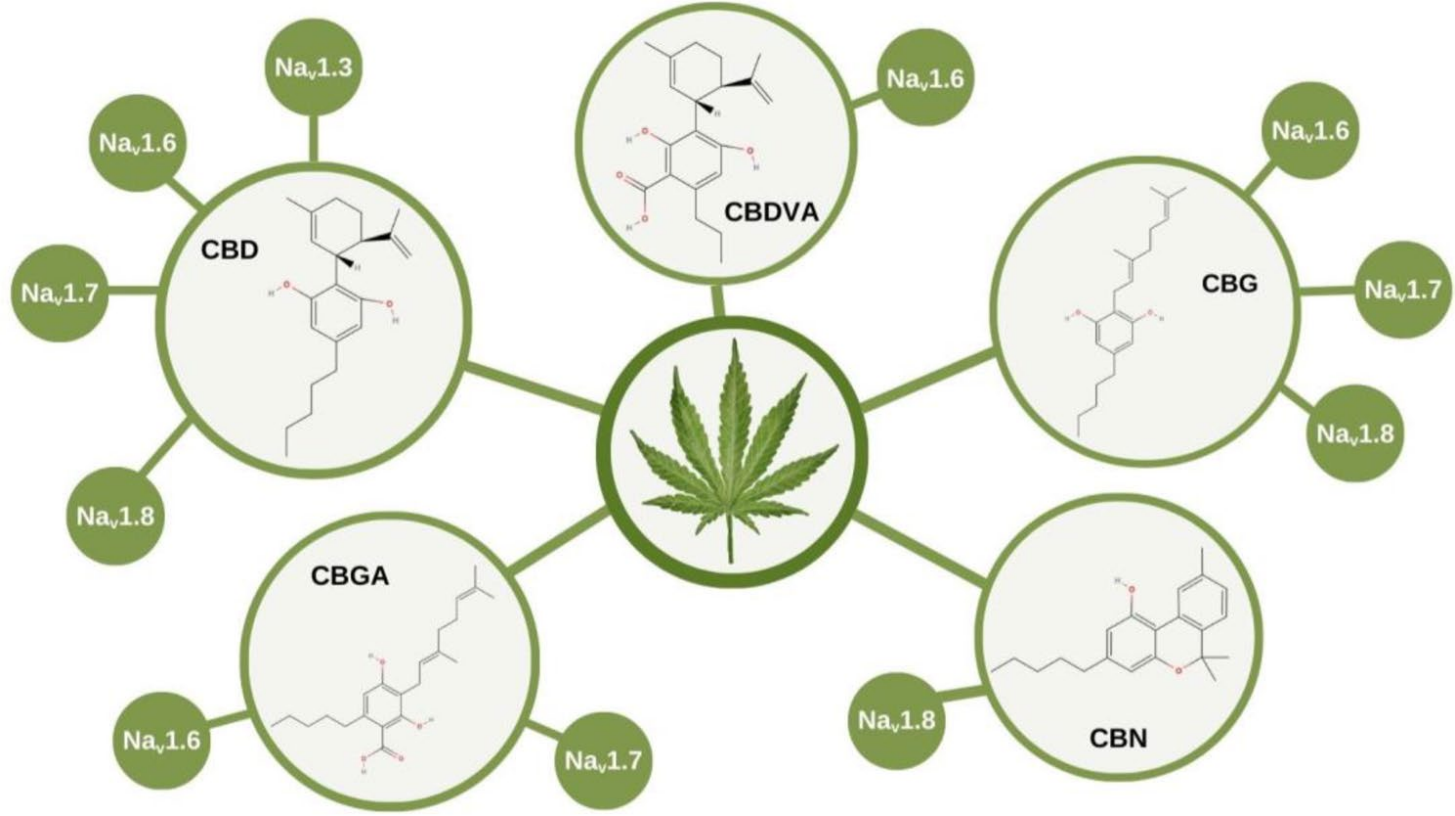
Chemical structures of selected cannabinoids and their modulatory effects on voltage-gated sodium channels (Na_v_). Cannabidiol (CBD), cannabidivarinic acid (CBDVA), cannabigerol (CBG), cannabinol (CBN), and cannabigerolic acid (CBGA) are shown with their reported inhibitory activity on Na_v_ subtypes

Cannabidiol (CBD) and derivatives, such as cannabigerol (CBG), cannabinol (CBN), cannabigerolic acid (CBGA), and cannabidivarinic acid (CBDVA), showed multiple electrophysiological effects on different Na_v_ involved on nociception (Table 1), as summarized in the Fig. 2.

**Table 1:** Electrophysiological and pharmacological outcomes of cannabinoids on sodium channels of the included manuscripts

Reference	Substance	Cell type	Method	Channel	Outcome	
Patel et al. 2016	CBD 1 µM (Cayman Chemical)	Rat striatal neurons	Whole-cell patch clamp	All	**IC**_**50**_	⊗
					**Voltage Clamp**	**Peak**: = **Resurgent**: ↓ * **ΔVg**_**1/2**_: = **ΔVh**_**1/2**_: -4.1 mV * **Recovery from inactivation (-80 mV)**: Ctrl: τ = 4.4 ± 0.3 ms CBD: τ = 7.5 ± 1.0 ms *
					**Current Clamp − 60 mV**	**AP firing**: ↓ (200 ms stimulus) * **AP peak**: ↓ 57% * **AP width**: ⊗ **AP threshold**: ↑ 32% *
					**Current Clamp − 80 mV**	**AP firing**: ↓ (200 ms stimulus) * **AP peak**: = **AP width**: ⊗ **AP threshold**: =
		HEK293	Whole-cell patch clamp	Nav 1.6 (NP_055006.1)	**Voltage Clamp**	**Peak**: = **Resurgent**: ↓ **ΔVg**_**1/2**_: ⊗ **ΔVh**_**1/2**_: ⊗ **Recovery from inactivation**: ⊗
Ghovanloo et al. 2018	CBD (Cayman Chemical)	HEK293	Whole-cell patch clamp	hNav1.3 (AF225987); hNav1.6 (NM_014191); mNav1.6 (NM_001077499); hNav1.7 (NM_002977)	**IC**_**50**_	hNav1.3: 3.3 ± 0.1 µM hNav1.6: 3.0 ± 0.1 µM mNav1.6: 2.4 ± 0.1 µM hNav1.7: 2.9 ± 0.1 µM
					**Voltage Clamp** **(HP = -120 mM)**	**Peak**: ↓ **Resurgent**: ↓ (hNav1.6) **ΔVg**_**1/2**_: ⊗ **ΔVh**_**1/2**_: ⊗ **Recovery from inactivation hNav1.6** (300 ms pre-pulse): Control: τ_fast_ = 1.73 ms; τ_slow_ = 68.8 ms CBD 3.7 µM: τ_fast_ = 6.54 ms; τ_slow_ = 516 ms **Recovery from inactivation hNav1.6** (10 s pre-pulse): Ctrl: τ_fast_ = 71.5 ms; τ_slow_ = 696 ms CBD 3.7 µM: τ_fast_ = 272 ms; τ_slow_ = 8.72 s
		iPSC neurons	Whole-cell automated patch-clamp	All	**Voltage Clamp**	**ΔVg**_**1/2**_: ⊗ **ΔVh**_**1/2**_: ↓ (1 µM CBD: -16 mV)
					**Current Clamp (model)**	**AP firing**: ↓ **AP peak**: ↓ **AP width**: ↑ **AP threshold**: ⊗
Zhang and Bean 2021	CBD (Sigma-Aldrich)	DRG neurons	Whole-cell patch clamp	rNav1.8	**IC**_**50**_	⊗
					**Voltage Clamp**	**Peak**: ↓ (85% − 5 µM – HP = -70 mV) **Ressurgent**: ⊗ **ΔVg**_**1/2**_: ⊗ **ΔVh**_**1/2**_: CBD 5 µM CBD (50 ms conditioning pulse) = -7.6 ± 0.4 mV CBD (200 ms conditioning pulse) = -10.6 ± 0.5 mV CBD (5 s conditioning pulse) = -15.7 ± 0.6 mV **Recovery from inactivation**: CBD 5 µM (HP = -70 mV): Ctrl (20 ms pre-pulse): τ_fast_ = 1.03 ms (max. 85%) CBD (20 ms pre-pulse): τ_fast_ = 1.03 ms (28%); τ_slow_ = 66 ms (72%) (max 80%) Ctrl (300 ms pre-pulse): τ_Dominant_ = 730 ms (max. 93%) CBD (300 ms pre-pulse): τ _Dominant_ = 2.03 s Ctrl (10 s pre-pulse): τ_Dominant_ = 2.72 s CBD (10 s pre-pulse): τ _Dominant_ = 9.57 s
					**Current Clamp** 2 µM CBD	**AP firing**: ↓ (88%) **AP peak**: ↓ (31.7%) **AP width**: ↑ (66%) **AP threshold**: ↑ (+ 9.5 mV)
Ghovanloo et al. 2022	CBG (Cayman Chemical)	HEK293	Whole-cell patch-clamp	hNav1.7 + β1	**IC**_**50**_	HP = -110 mV: 18.8 ± 2.9 µM HP = -100 mV: 9.3 ± 1.0 µM HP = -90 mV: 4.6 ± 1.1 µM
					**Voltage Clamp**	**Peak**: ↓ * (15 µM: ~90%) **Ressurgent**: ⊗ **ΔVg**_**1/2**_: CBG 4 µM: = CBG 15 µM: = **ΔVh**_**1/2**_ (hyperpolarization 500 ms): CBG 4 µM: = CBG 15 µM: -17.2 mV* **Recovery from inactivation −** 500 ms pre-pulse: Control: τ_fast_ = 5.0 ± 0.3 ms; τ_slow_ = 154 ± 16 ms CBG 4 µM: τ_fast_ = 6.7 ± 0.5 ms; τ_slow_ = 217 ± 21 ms CBG 15 µM: τ_fast_ = 60.4 ± 4.7 ms; τ_slow_ = 936 ± 173 ms **Recovery from inactivation −** 5 s pre-pulse: Control: τ_fast_ = 5.5 ± 0.6 ms; τ_slow_ = 280 ± 16 ms CBG 4 µM: τ_fast_ = 4.3 ± 0.9 ms; τ_slow_ = 832 ± 58 ms CBG 15 µM: τ_fast_ = 87 ± 26 ms; τ_slow_ = 1.07 ± 0.12 s
				TTX-R Na^+^ channels	**Voltage Clamp**	**Peak**: ↓ (10 µM - ~50%)
		DRG neurons	MEA	All	**Current Clamp**	**AP firing**: ↓* (2 µM: 32%; 15 µM: 89%) **AP peak**: ↓ # **AP width**: ⊗ **AP threshold**: ⊗
Milligan et al. 2023	CBD (THCPharm), CBGA (Invizyme), CBDVA (Prof. Michael Kassiou, University of Sidney)	CHO	Whole-cell automated patch-clamp	hNav1.6 hNav1.7	**IC**_**50**_	*hNav1.6*: CBD: 16.6 ± 1.8 µM CBGA: 12.0 ± 1.2 µM CBDVA: 24.1 ± 1.2 µM *hNav1.7*: CBD: 11.9 ± 2.2 µM CBGA: 16.4 ± 1.1 µM CBDVA: ≥ 60 µM
					**Voltage Clamp**	**Peak**: ↓* **Ressurgent**: ⊗ **ΔVg**_**1/2**_: *hNav1.6*: CBD: -0.6 ± 1.1 mV CBGA: 0.1 ± 1.6 mV CBDVA: 10.4 ± 2.9 mV* *hNav1.7*: CBD: 5.7 ± 2.3 mV* CBGA: 4.6 ± 1.7 mV* CBDVA: 8.7 ± 1.8 mV* **ΔVh**_**1/2**_: *hNav 1.6*: CBD: -5.3 ± 3.6 mV CBGA: -0.5 ± 2.4 mV CBDVA: -3.0 ± 1.3 mV* *hNav 1.7*: CBD: -3.7 ± 1.2 mV* CBGA: -5.5 ± 2.1 mV* CBDVA: -4.1 ± 0.9 mV* **ΔRecovery from inactivation**: *hNav1.6*: CBD: 0.3 ± 0.1 ms* CBGA: 0.9 ± 0.4 ms CBDVA: 0.6 ± 0.1 ms* *hNav1.7*: CBD: 3.6 ± 0.6 ms* CBGA: 3.2 ± 0.8 ms* CBDVA: 1.6 ± 04 ms*
					**Current Clamp**	**AP firing**: ⊗ **AP peak**: ⊗ **AP width**: ⊗ **AP threshold**: ⊗
Huang et al. 2023	CBD (Sigma-Aldrich)	HEK293	Whole-cell patch clamp	hNav 1.7 (Q15858)	**IC**_**50**_	1.82 ± 0.1 µM
					**Voltage Clamp**	**Peak (CBD 300 nM)**: ↓* (71%; HP=-70 mV) **Ressurgent**: ⊗ **ΔVg**_**1/2**_: ⊗ **ΔVh**_**1/2**_: **(CBD 1 µM)** -9.26 ± 0.69 mV (50 ms prepulse) -11.5 ± 0.7 mV* (5 s prepulse) **Recovery from inactivation**: ↓ **#**
					**Current Clamp**	**AP firing**: ⊗ **AP peak**: ⊗ **AP width**: ⊗ **AP threshold**: ⊗
Ghovanloo et al. 2025	CBD, CBG, CBN	DRG neurons	Whole-cell automated patch-clamp	rNav1.8	**IC**_**50**_	CBD: ~2 µM CBG: ~5 µM CBN: ~16 µM
					**Voltage Clamp**	**Peak**: ↓* # **Ressurgent**: ⊗ **ΔVg**_**1/2**_: = (for all) **ΔVh**_**1/2**_ **(500 ms prepulse)**: CBD ↓* # CBG ↓* # CBN ↓* # **Recovery from inactivation**: CBD ↓* # CBG ↓* # CBN ↓* #
				All	**Current Clamp**	**AP firing**: ↓* (CBG 5 µM) **AP peak**: ↓* (CBG 5 µM) **AP width**: ⊗ **AP threshold**: ⊗

*CDB* Cannabidiol, *CBG* Cannabigerol, *CBGA* Phytocannabichromenic acid, *CBDVA* Cannabidivarinic acid, *CBN* Cannabinol, *HEK293* Human embryonic kidney cells, *DRG* Dorsal root ganglion, *MEA* Multi-electrode array, *AP* Action potential, *HP* Holding potential, *ΔVg1/2* Difference between the potential that activates 50% of the maximal conductance in the presence of the drug and in the control condition, *ΔVh1/2* Difference between the potential that inactivates 50% of the channels in the presence of the drug and in the control condition, ↑ - increased, ↓ - decreased/hyperpolarize, = not induce/no effect, ⊗ – not made/not measured, # - no numbers were presented in the manuscript

*p < 0,05

Electrophysiological protocols analyzed in this review for voltage clamp experiments include: (i) voltage dependence of activation (Vg_1/2_), which reflects the voltage at which half of the channels transition from the closed to the open state; (ii) steady-state inactivation (Vh_1/2_), which evaluates the conditioning voltage that inactivates half of the channels and characterizes the transition from closed to inactivated states — a process that can occur without channel opening; and (iii) recovery from inactivation, which measures the time required during hyperpolarization for channels to transition from the inactivated to the available (closed) state.

In rat striatal neurons, CBD (1 µM) prolonged recovery from inactivation and significantly reduced resurgent currents, while in current-clamp mode (holding potential, HP = -80 mV) it decreased action potential (AP) firing frequency, reduced AP peak amplitude, and increased AP threshold. For more hyperpolarized HP, these effects were mitigated, but the inhibition of AP firing. Comparable effects were observed in HEK293 cells expressing Na_v_1.6, where CBD reduced resurgent currents without altering peak currents (Patel et al. 2016).

CBD inhibited multiple human and murine sodium channel isoforms (hNa_v_1.3, hNa_v_1.6, hNa_v_1.7, mNa_v_1.6) with IC_50_ values ranging from 2.4 to 3.3 µM. Notably, CBD prolonged recovery from inactivation of hNa_v_1.6, indicating strong use-dependent inhibition. Similarly, in induced pluripotent stem cell (iPSC)-derived neurons, CBD hyperpolarized Vh_1/2_ (–16 mV at 1 µM) and reduced AP firing, consistent with a stabilizing effect on channel inactivation (Ghovanloo et al. 2018).

In DRG neurons were reported that CBD (5 µM) produced a strong reduction in Na_v_1.8 peak currents (85%), with progressive hyperpolarization of Vh_1/2_ depending on conditioning pulse duration. Recovery from inactivation was markedly slowed, and at the cellular level, CBD reduced AP firing by ~ 88%, decreased AP peak amplitude, broadened AP width, and shifted AP threshold to more depolarized values (Zhang and Bean 2021).

Other phytocannabinoids also demonstrated sodium channel inhibition. Cannabigerol (CBG) inhibited hNa_v_1.7 currents in a voltage- and holding potential–dependent manner (IC_50_ values between 4.6 and 18.8 µM). CBG reduced peak sodium currents and shifted Vh_1/2_ to more hyperpolarized potentials at higher concentrations (15 µM). In DRG neurons, CBG dose-dependently decreased AP firing, with up to ~ 89% inhibition at 15 µM (Ghovanloo et al. 2022). Milligan and coworkers (2023) found that CBD and CBGA inhibited hNa_v_1.6 and hNa_v_1.7 channels with micromolar potency (IC_50_: 11.9–16.6 µM), however they shifted activation and inactivation parameters only in hNa_v_1.7 channels. Although CBDVA inhibited the channels with lower affinity (IC_50_: 24.1 and ≥ 60 µM for hNa_v_1.6 and hNa_v_1.7, respectively), it significantly modified the biophysical parameters of both channels, depolarizing Vg_1/2_, hyperpolarizing Vh_1/2_, and slowing inactivation recovery kinetics (Milligan et al. 2023).

CBD potently inhibits hNa_v_1.7 (IC₅₀ ≈ 1.8 µM), accompanied by a marked hyperpolarizing shift in Vh_1/2_ (up to − 11.5 mV at 1 µM) (Huang et al. 2023). CBD, cannabigerol (CBG), and cannabinol (CBN) on Na_v_1.8 channels in DRG neurons inhibited peak currents, induced a negative shift in Vh_1/2_, and slowed recovery from inactivation, with CBD showing the highest potency (IC₅₀ ≈ 2 µM). Functionally, CBG reduced action potential firing and peak amplitude at micromolar concentrations (Ghovanloo et al. 2025).

## Discussion

Cannabidiol and derivatives act in several voltage-gated sodium channels (Table 1), and two principal mechanisms have been proposed to explain its inhibitory effects: (i) direct blockade of channel conductance and (ii) stabilization of the inactivated state. Given its high lipophilicity, CBD is likely to accumulate within the plasma membrane rather than in extracellular or cytosolic compartments.

### Voltage-gated sodium channel family (Na_v_)

**Fig. 3:**
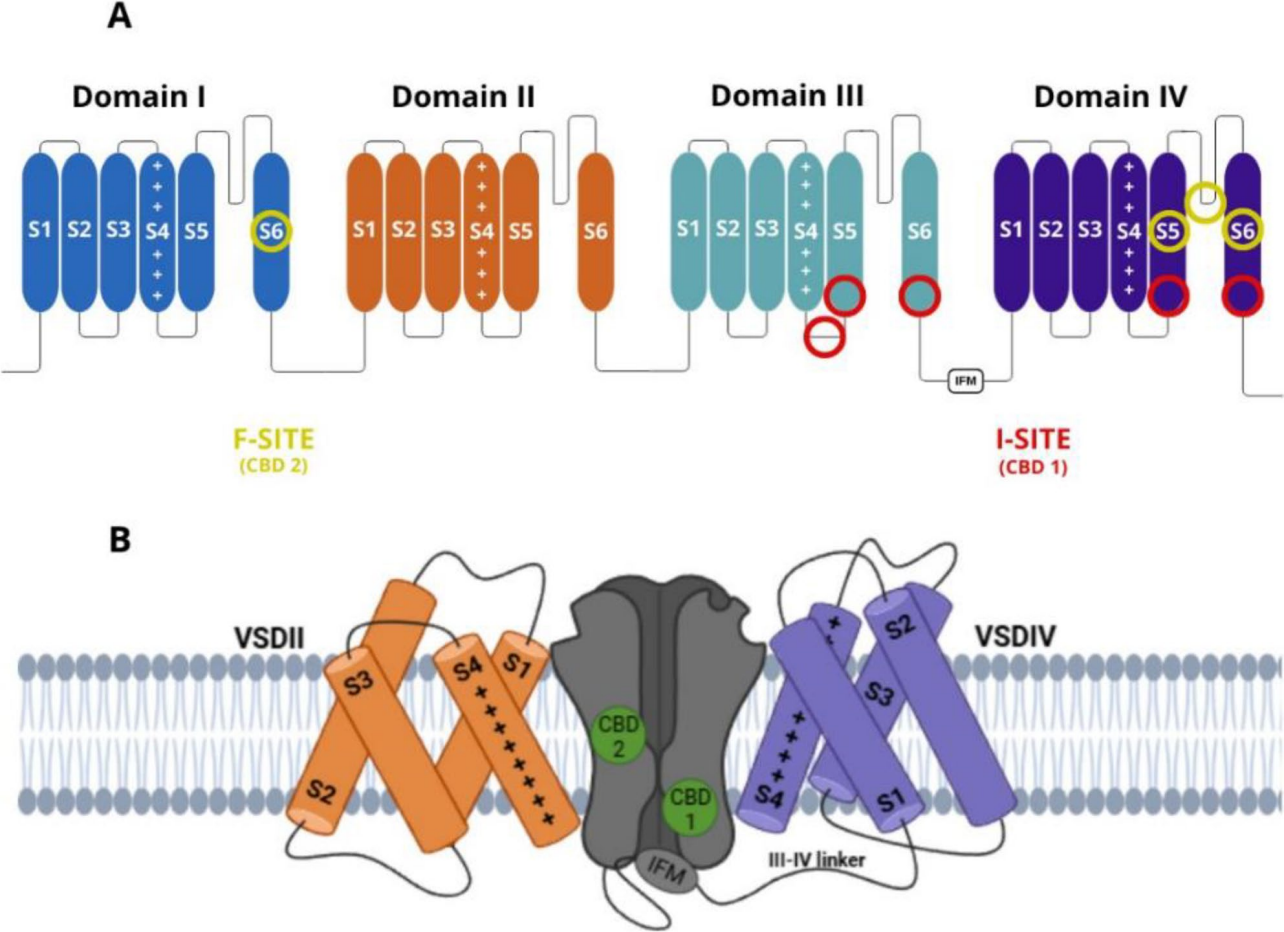
Structural representation of cannabidiol (CBD) binding sites within the voltage-gated sodium channel (Na_v_). (**a**) Schematic representation of the four transmembrane domains (I–IV) of voltage-gated sodium channels (Na_v_), each containing six transmembrane segments (S1–S6). The voltage-sensing domains (S1–S4) are indicated, with the positively charged S4 helices highlighted. The pore-lining S5–S6 segments form the central conduction pathway. Functional binding regions are marked: the I-site (CBD-1) in domains III and IV (red), and the F-site (CBD-2) in domain I and IV (yellow); which are associated with the modulation of channel activity and represent potential pharmacological interaction sites. (**b**) Interaction of CBD molecules (green) at two distinct binding pockets (CBD1 and CBD2) located between the pore domain and the voltage-sensing domains VSDII (orange) and VSDIV (purple). The intracellular III–IV linker and IFM motif, critical for fast inactivation, are also depicted. This arrangement highlights the potential mechanism by which CBD modulates Na_v_ channel gating and inactivation (inspired by Huang et al. 2023)

Voltage-gated sodium (Na_v_) channels are protein structures found in excitable cells, playing a crucial role in modulating electrical potential by regulating sodium ion influx. This channel family comprises nine subtypes, designated numerically (Na_v_1.1 to Na_v_1.9). Each channel consists of an α-subunit containing four homologous domains, each with six transmembrane segments, and can interact with a β-subunit, which modified its biophysics properties (Fig. 3A).

### Electrophysiological effects of CBD on Na_v_1.3 channels

The Na_v_1.3 sodium channel is encoded by the SCN3A gene. These channels are abundantly expressed in the embryonic and neonatal central nervous system but are scarce in the normal adult brain. They have also been identified in the transverse tubules of cardiomyocytes (Catterall et al. 2005). Following peripheral axotomy, the expression of Na_v_1.3 is upregulated in adult DRG neurons, as well as in the dorsal horn of the spinal cord and the thalamus (Heiland et al. 2023). The contribution of Na_v_1.3 to neuropathic pain is believed to be linked to increased hyperactivity in sensory neurons, which results in overall hyperexcitability and a lowered nociceptive threshold. This involvement has been further confirmed by the attenuation of neuropathic pain following intraganglionic injection of an adenovirus expressing a small hairpin RNA designed to silence Na_v_1.3 expression specifically in nociceptive neurons (Liao et al. 2023; Samad et al. 2013).

CBD achieves complete blockade of Na_v_1.3 at concentrations above 10 µM (Ghovanloo et al. 2018). In contrast, nutraceutical product (NP) exhibited an IC_50_ of 4.0 ± 0.2 µg/mL, corresponding to 233 nM of CBD (Milligan et al. 2022). The relatively lower blocking efficiency of NP compared to pure CBD may be attributed to a putative synergistic effect of other compounds. Additionally, NP statistically shifted the Vg_1/2_ and Vh_1/2_ toward hyperpolarized values by -5.3 mV and − 4.4 mV, respectively.

### Electrophysiological effects of cannabinoids on Na_v_1.6 of neurons

The Na_v_1.6 sodium channel is encoded by the SCN8A gene. Initially identified in the central nervous system of rats, this channel is highly expressed and widely distributed. It is found in both excitatory and inhibitory neurons, glial cells, pyramidal and granule cells of the hippocampus, motor neurons in the spinal cord and brainstem, axons in the retina, and dendrites of cortical pyramidal cells (Catterall et al. 2005). This channel mediate persistent and resurgent sodium currents that contribute to motor neuron activity in mice (Solé and Tamkun 2020), and enhance sensory neuron excitability in the DRG (Xie et al. 2015). Na_v_1.6 channels are clustered primarily at the nodes of Ranvier in myelinated fibers, where they play a critical role in action potential generation and fast saltatory conduction, as well as continuous conduction in unmyelinated axons (Bennett et al. 2019). Local knockdown of Na_v_1.6 using siRNA in animal models blocks pain behaviors associated with inflammation and nerve injury (Xie et al. 2013).

Electrophysiological study demonstrated that CBD (1 µM) did not significantly inhibit the peak current of Na_v_1.6 but effectively reduced resurgent currents (Patel et al. 2016). However, other studies reported significant peak current inhibition (Ghovanloo et al. 2018; Milligan et al. 2023). The discrepancy in IC_50_ values may be attributed to the expression system (HEK293 and CHO cells respectively) or differences in CBD purity across studies, since they were acquired from different distributors. Additionally, CBD significantly shifted the Vh_1/2_ toward more hyperpolarized potentials (Patel et al. 2016). Regarding inactivation recovery, 3.7 µM CBD delayed the transition from inactivated to closed state, following a biexponential decay in which the slower time constant component was associated with CBD-bound channels (Ghovanloo et al. 2018). Although the inactivation recovery curve fits a single exponential, the decay was also significantly slower in the presence of 1 µM CBD (Patel et al. 2016).

Na_v_1.6 peak currents were inhibited by NP with an IC_50_ of 1.7 ± 0.3 µg/mL, equivalent to 99 nM CBD. Channel activation voltage dependence remained unchanged; however, 3 µg/mL NP significantly shifted Vh_1/2_ toward more hyperpolarized potentials (control: -52.2 ± 2.4 mV; NP: -58.7 ± 3.1 mV), and delayed inactivation recovery, reinforcing the role of synergism among the components (Milligan et al. 2022).

Different phytocannabinoids also modulated this channel. Milligan and collaborators (2023) reported that among the tested compounds, CBDVA inhibited Na_v_1.6 currents and positive shifted Vg_1/2_ while negative shifting Vh_1/2_. CBGA exhibited higher affinity but did not alter the biophysical parameters of the channel. Other phytocannabinoids (CBG, cannabichromenic acid - CBCA, and cannabichromene - CBC) showed no significant effects on Na_v_1.6 currents (Milligan et al. 2023).

Interestingly, the potency and kinetics of CBD-mediated inhibition of hNa_v_1.6 are enhanced at lower temperatures, suggesting that this interaction may not occur through a single, well-defined binding site on the channel (Ghovanloo et al. 2018). Based on the steep Hill slope consistently observed across many experiments, it becomes evident that the binding mechanism does not follow the expected one-to-one interaction (Milligan et al. 2023).

### Mechanistic insights into Na_v_1.7 inhibition by cannabidiol and phytocannabinoids

The sodium channel Na_v_1.7 is encoded by the SCN9A gene. These channels were initially identified in the involuntary nervous system, specifically in DRG neurons and sympathetic ganglia (Catterall et al. 2005). Upon stimulation of presynaptic neuronal terminals, a transient membrane depolarization occurs, which is amplified by the opening of Na_v_1.7 channels until reaching the threshold, ultimately triggering neuronal firing (Trombetti et al. 2022).

Since three Na_v_1.7 mutations were associated with congenital insensitivity to pain, significant efforts have been devoted to developing selective blockers for this channel (Cox et al. 2006). However, due to structural similarities among Na_v_ isoforms, careful investigation is required to prevent cross-reactivity, which could lead to severe adverse effects (Trombetti et al. 2022). It has been proposed that analgesia resulting from Na_v_1.7 deletion occurs only when neurotransmitter release is inhibited, and the absence of Na_v_1.7 does not affect peripheral excitability. Instead, it is associated with a marked reduction in synaptic transmission from central nociceptors in the spinal cord (Cox et al. 2006).

Electrophysiological experiments with cells expressing Na_v_1.7 channels revealed that CBD induced modifications was not different in the presence of the accessory subunits β1 and β2 (Huang et al. 2023).

Combining electrophysiology and cryo-electron microscopy, CBD binds to two distinct sites on the Na_v_1.7 channel, neither of which directly occludes the pore, instead, CBD inhibits the channel allosterically (Fig. 3a) (Huang et al. 2023). The authors proposed that binding at site 1 (I-site), located in the loop between domains III and IV near the IFM motif, alters the structure of the S6 segment in domain III, stabilizing the channel in the inactivated state (Fig. 3b). This finding explains the hyperpolarizing shift in Vh_1/2_ and the slowed recovery from inactivation upon membrane repolarization. Given CBD’s lipophilic nature, site 2 (F-site) is hypothesized to reside within hydrophobic fenestrations of the channel, possibly between domains IV and I (Fig. 3a and b). Since this fenestration does not extend to the pore, it is unlikely to contribute to direct pore blockade (Huang et al. 2023).

CBGA caused a significant depolarization of the activation curve, a hyperpolarizing shift in Vh_1/2_, and a slowing of both inactivation kinetics and recovery from inactivation (Milligan et al. 2022, 2023). Similarly, CBDVA induced a strong depolarization of activation voltage, a hyperpolarizing shift in Vh_1/2_, and an increase in its slope. Moreover, CBDVA slowed the recovery from inactivation. They revealed that other phytocannabinoids (CBG, CBCA, and CBC) exhibited modest inhibitory effects on Na_v_1.7 currents. However, other authors reported that CBG blocks the Na_v_1.7 channel. The mechanism involves inhibition of conductance at lower concentrations (IC₅₀ = 3.5 µM) and a hyperpolarizing shift of Vh_1/2_ at higher concentrations (IC₅₀ = 13.2 µM), suggesting that conductance blockade is the most relevant effect in reducing sodium channel activity associated with pain (Ghovanloo et al. 2022).

The entourage effect of NP (3 µg/mL) inhibited Na_v_1.7 channel, reducing the peak current from 1.9 ± 0.3 nA in the control condition to 1.2 ± 0.2 nA in the presence of NP, with an IC_50_ of 1.6 ± 0.1 µg/mL. NP treatment induced a hyperpolarizing shift Vh_1/2_ (ΔVh_1/2_ = -3.8 mV). Furthermore, inactivation recovery was significantly slower, with time constants of 2.7 ± 0.2 ms in the control and 3.8 ± 0.5 ms in the presence of NP (Milligan et al. 2022).

### Phytocannabinoids on action potential upstroke – the molecular role of Na_v_1.8

The voltage-gated sodium channel Na_v_1.8, encoded by the SCN10A gene, is characterized by its resistance to tetrodotoxin (TTX), a classical sodium channel blocker. Na_v_1.8 is expressed in a subset of sensory neurons within the DRG and plays a pivotal role in nociceptive neurotransmission. Approximately 75% of DRG neurons are estimated to express this channel (Akopian et al. 1999). Compared with other isoforms, Na_v_1.8 displays a more depolarized activation threshold, slower inactivation kinetics, and a characteristic persistent current (Miller et al. 2017). The low efficacy of Na_v_1.8 expression in heterologous systems has hindered the functional characterization of CBD and its derivatives in immortalized cell lines; consequently, no studies have yet reported Na_v_1.8 expression with cannabinoids in such systems. Instead, functional isolation of this channel has only been achieved in DRG neurons from Na_v_1.9^−/−^ mice, using 500 nM TTX to block all TTX-sensitive Na_v_ channels (Ghovanloo et al. 2025).

As expected for a compound that blocks nociceptive signaling, CBD (2 µM) and CBG (5 µM) reduced the firing frequency of action potentials in DRG neurons (Zhang and Bean 2021; Ghovanloo et al. 2025). This effect was associated with decreased spike amplitude, prolonged action potential duration, and membrane depolarization. The underlying mechanism involved in CBD inhibition of Na_v_1.8 currents is characterized by preferential binding to the inactivated state and a frequency-dependent profile, resulting in both tonic and use-dependent inhibition under near physiological stimulation. In addition, CBD slowed channel inactivation kinetics and introduced an additional slow component in recovery from inactivation, indicating impaired transition from the inactivated to the closed state. Moreover, CBD and other cannabinoids (CBG and CBN) shifted Vh_1/2_ of Na_v_1.8 channels toward more hyperpolarized potentials (Zhang and Bean 2021), particularly under conditions favoring slow inactivation (Zhang and Bean 2021; Ghovanloo et al. 2025). Although CBG exhibited a higher IC_50_ than CBD, CBG exerted the strongest inhibitory effect on the biophysical parameters among the cannabinoids tested (CBD, CBG, and CBN). Collectively, these findings suggest that cannabinoids stabilizes the slow inactivated state of Na_v_1.8 channels, thereby attenuating repetitive firing in nociceptive neurons (Zhang and Bean 2021; Ghovanloo et al. 2025).

These channels were not affected by 3 µg/mL of NP; however, a statistically significant shift was observed in the activation (ΔVg_1/2_ = − 6.9 mV) and in the steady-state inactivation voltage (ΔVh_1/2_ = − 7.2 mV) (Milligan et al. 2022).

### Findings about cannabinoids effects on Na_v_1.9

The SCN11A gene encodes the Na_v_1.9 channel, which is characterized by resistance to TTX and is preferentially expressed in nociceptive neurons, such as those of the DRG and trigeminal ganglia (Catterall et al. 2005). Biophysically, Na_v_1.9 activates and inactivates within a hyperpolarized voltage range with slow kinetics, generating a persistent, low-threshold current that amplifies subthreshold depolarizations (Dib-Hajj et al. 2010). Due to the difficulty of expressing Na_v_1.9 in heterologous systems, few studies have examined this channel in isolation, and none have investigated its interaction with cannabinoids. However, the effects of an endocannabinoid analogue of anandamide on an hNa_v_1.9/rNa_v_1.4 chimera, showing features similar to cannabinoid actions on nociceptive Na_v_ channels, including use-dependent channel block, a negative shift in Vh_1/2_, state-dependent binding affinity (preferential binding to the inactivated state), besides slower fast inactivation kinetics (Marchese-Rojas et al. 2023). These findings suggest that cannabinoids may exert a similar blocking mechanism on Na_v_1.9, although this remains to be clarified.

## Conclusion

Cannabinoids act as non-selective inhibitors of voltage-gated sodium channels, a key mechanism in nociceptive signaling. A consistent observation is their ability to reduce sodium current and shift the half-maximal voltage for steady-state inactivation (Vh_1/2_) to more hyperpolarized potentials, effects that are enhanced by longer depolarization steps. While cannabidiol (CBD) preferentially binds to the inactivated state of these channels, cannabigerol (CBG) appears to reduce maximal conductance (G_Max_) at lower concentrations, a mechanism that may be more clinically relevant. At the cellular level, these actions collectively decrease neuronal excitability, reducing the frequency of action potential firing, which aligns with their reported efficacy in treating conditions like epilepsy and pain.

This inhibitory effect is not limited to isolated compounds, as the synergistic action of multiple cannabinoids, terpenes, and flavonoids present in cannabis-derived products — also plays a crucial role. For instance, minor constituents such as CBG, CBDVA, and CBGA have been shown to modulate sodium channels.

## Data Availability

No datasets were generated or analysed during the current study.

## Abbreviations

CDB: Cannabidiol
CBG: Cannabigerol
CBGA: Phytocannabichromenic acid
CBDVA: Cannabidivarinic acid
CBN: Cannabinol
HEK293: Human embryonic kidney cells
DRG: Dorsal root ganglion
MEA: Multi-electrode array
AP: Action potential
HP: Holding potential
ΔVg_1/2_: Difference between the potential that activates 50% of the maximal conductance in the presence of the drug and in the control condition
ΔVh_1/2_: Difference between the potential that inactivates 50% of the channels in the presence of the drug and in the control condition
↑: Increased/depolarize
↓: Decreased/hyperpolarize
=: Not induce/no effect
⊗: Not made/not measured
#: No numbers were presented in the manuscript
